# Comparison of In Vivo and Ex Vivo Magnetic Resonance Imaging in a Rat Model for Glioblastoma-Associated Epilepsy

**DOI:** 10.3390/diagnostics11081311

**Published:** 2021-07-21

**Authors:** Charlotte Bouckaert, Emma Christiaen, Jeroen Verhoeven, Benedicte Descamps, Valerie De Meulenaere, Paul Boon, Evelien Carrette, Kristl Vonck, Christian Vanhove, Robrecht Raedt

**Affiliations:** 1 4Brain, Department of Head and Skin, Ghent University, 9000 Ghent, Belgium; charlotte.bouckaert@ugent.be (C.B.); paul.boon@ugent.be (P.B.); evelien.carrette@ugent.be (E.C.); kristl.vonck@ugent.be (K.V.); 2 Department of Electronics and Information Systems, Ghent University, 9000 Ghent, Belgium; emma.christiaen@ugent.be (E.C.); benedicte.descamps@ugent.be (B.D.); christian.vanhove@ugent.be (C.V.); 3 Department of Diagnostic Sciences, Ghent University, 9000 Ghent, Belgium; jeroen.verhoeven@ugent.be; 4 Department of Radiology, Ghent University, 9000 Ghent, Belgium; valerie.demeulenaere@ugent.be

**Keywords:** glioblastoma, in vivo MRI, ex vivo MRI, peritumoral edema, mean diffusivity

## Abstract

Magnetic resonance imaging (MRI) is frequently used for preclinical treatment monitoring in glioblastoma (GB). Discriminating between tumors and tumor-associated changes is challenging on in vivo MRI. In this study, we compared in vivo MRI scans with ex vivo MRI and histology to estimate more precisely the abnormal mass on in vivo MRI. Epileptic seizures are a common symptom in GB. Therefore, we used a recently developed GB-associated epilepsy model from our group with the aim of further characterizing the model and making it useful for dedicated epilepsy research. Ten days after GB inoculation in rat entorhinal cortices, in vivo MRI (T2w and mean diffusivity (MD)), ex vivo MRI (T2w) and histology were performed, and tumor volumes were determined on the different modalities. The estimated abnormal mass on ex vivo T2w images was significantly smaller compared to in vivo T2w images, but was more comparable to histological tumor volumes, and might be used to estimate end-stage tumor volumes. In vivo MD images displayed tumors as an outer rim of hyperintense signal with a core of hypointense signal, probably reflecting peritumoral edema and tumor mass, respectively, and might be used in the future to distinguish the tumor mass from peritumoral edema—associated with reactive astrocytes and activated microglia, as indicated by an increased expression of immunohistochemical markers—in preclinical models. In conclusion, this study shows that combining imaging techniques using different structural scales can improve our understanding of the pathophysiology in GB.

## 1. Introduction

Glial tumors or gliomas represent 80% of all malignant brain tumors [1,2]. Grade IV gliomas, also called glioblastoma (GB), represent the most common and aggressive form of malignant primary brain tumors in adults, with an average age-adjusted incidence of 3.2 cases per 100,000 people [3]. GB account for approximately 15% of all central nervous system tumors and approximately 50% of all gliomas [1,2,4]. The current standard of care for newly diagnosed GB patients consists of maximum safe surgical resection, followed by radiation therapy, with concomitant and adjuvant chemotherapy with temozolomide [5]. Despite multimodal aggressive therapy, the prognosis for GB patients remains poor with a median survival of 15–17 months and a 5-year survival rate of 5% [6,7]. Relapse occurs in the cavity margin in 85% of all cases and, therefore, GB remains an essentially incurable disease [8,9].

Seizures represent a common symptom in GB and occur in 30–60% of patients with GB [10,11]. Up to 30% of patients with GB suffer from drug-resistant epilepsy (DRE), resulting in significant morbidity and a negative impact on quality of life [10,12,13,14].

Better treatments are needed, and the search for new therapies requires the use of animal models that mimic disease progression in humans. Several rodent models for GB have been developed [15]. The syngeneic Fischer/F98 rat model of GB, characterized as a GB-related epilepsy model [16] at Ghent University, has characteristics that closely resemble those of human GB with regard to its aggressiveness, histological appearance and lack of immunogenicity [17], and this model was used in the current study.

Magnetic resonance imaging (MRI) plays a crucial role in diagnosis and treatment monitoring. As optimal surgical resection or radiation therapy of tumors requires an exact knowledge of the spatial extent of tumor growth, the main goal of non-invasive imaging in brain tumor diagnosis consists of the tumor localization, determination of tumor extent and detection of brain tissue infiltrated by the tumor. Therefore, it is important to distinguish between tumor tissue and tumor-related reversible changes in normal brain tissue, such as edema [18]. MRI with (contrast-enhanced) T1-weighted (T1w), T2-weighted (T2w) and/or fluid-attenuated inversion recovery (FLAIR)-weighted sequences serves as the current gold standard in tumor treatment response monitoring, by assessing the location and extent of the tumor before, during, and after treatments [19]. Abnormal regions highlighted in the MR images depend not only on the tumor cells but also on the tumor microenvironment (TME), particularly the vasculature that results primarily from a compromised blood-brain barrier (BBB) [20]. Peritumoral brain edema is frequently seen in GB patients and can be visualized on MRI as a region of increased T2 signal intensity at the tumor margin. However, there is no consensus for the measurement or classification of peritumoral edema on MRI, and the appearance of edema and tumor mass on MRI is variable [21,22]. Laboratory animal imaging requires a higher field strength, in the range of 4.7–11.7 T (most commonly 7 T), compared to the standard clinical range of 1.5–3 T to obtain a better spatial resolution. High-field scanners result in an increased signal-to-noise ratio (SNR), but also in increased image artifacts and other limitations, making these higher field-strength scans technically more challenging to interpret [23]. Therefore, distinguishing between edema (and other tumor-related brain tissue changes) and tumor mass is even more difficult in preclinical animal models.

Diffusion-weighted imaging (DWI) is an advanced MRI technique with a high sensitivity to changes in the microscopic cellular environment, without the need for contrast injection [24]. The apparent diffusion coefficient (ADC), a metric derived from DWI, reflects the volume of the extracellular water compartment. ADC is sensitive to changes in cell density, edema and necrosis [25,26]. Diffusion tensor imaging (DTI) is developed from DWI and uses additional magnetic gradients in at least six directions to create a model of diffusion in three dimensions. In DTI, the full diffusion tensor (DT) describing the degree of anisotropic water diffusion in the tissue using a Gaussian model is measured. This DT is a 3 × 3 symmetric, positive–definite matrix with three orthogonal eigenvectors and three positive eigenvalues. The average of the tensor’s eigenvalues is called the mean diffusivity (MD), and this parameter relates to the total amount of diffusion in a voxel and, thus, to the amount of water in the extracellular space by measuring the diffusion magnitude or average anisotropy [24,27,28]. An increase in MD values has been shown in peritumoral edema surrounding high-grade gliomas compared to the contralateral white matter [28]. Decreased MD values might reflect high cellularity in the tumor mass, whereas increased MD values might also be seen in necrotic tumor areas apart from peritumoral edema [29].

Ex vivo MRI enables longer scan times and limits motion, boosting the resolution and SNR. However, distortions and dehydration from fixation, disrupting the integrity, will be observed, and longitudinal follow-up is ruled out [30,31]. Moreover, fixation has been shown to significantly lower water diffusivities, and detection of edema is precluded [32,33,34]. Therefore, ex vivo T2w images might be particularly interesting to identify tumor mass without the presence of peritumoral edema.

In this study, anatomical changes in the syngeneic Fischer/F98 rat model of GB were compared using in vivo MRI, ex vivo MRI and histology. Moreover, in vivo MD images and immunohistochemical markers were explored to identify the contribution of peritumoral edema, and an association with reactive astrocytes and activated microglia, to the estimated abnormal mass on MRI.

## 2. Materials and Methods

### 2.1. F98 Cells

F98 (ATCC^®^ CRL-2397™) rat GB cells were cultured as monolayers in Dulbecco’s Modified Eagle Medium (DMEM), supplemented with 10% fetal calf serum (FCS), 1% penicillin-streptomycin, 1% L-glutamine, and 0.1% fungizone (all products for cell culture were purchased from Invitrogen^®^ (Merelbeke, Belgium)) and placed in an incubator at 37 °C and 5% CO_2_.

### 2.2. Animals

Thirty male F344/IcoCrl rats (Charles River^®^) at 9–10 weeks old and with a mean bodyweight of 235 ± 14 g were used. The study was approved by the animal ethics committee of the Faculty of Medicine and Health Sciences of Ghent University (ECD 17/111). All animals were kept and handled according to European guidelines (Directive 2010/63/EU) and housed under environmentally controlled conditions: 12-h light/dark cycle, temperature between 21 and 24 °C, and humidity between 55 and 65%. Food and water were provided ad libitum.

### 2.3. Inoculation

All animals were anesthetized with a mixture of medical oxygen and isoflurane (induction: 5%; maintenance: 2%) and immobilized in a stereotaxic frame. Body temperature was maintained at 37 °C by a thermoregulated heating pad. The glioma cell suspension was stereotactically injected in the right entorhinal cortex (anteroposterior (AP): −8.0 mm and mediolateral (ML): +4.5 mm relative to bregma, dorsoventral (DV): −4.1 mm relative to dura) using an insulin syringe (BD 0.5 mL insulin syringe, microfine 0.33 mm (29G) × 12.7 mm) mounted on a pump for automatic injection (Stoelting Quintessential Stereotaxic Injector, Stoelting Co., Wood Dale, IL, USA). In all animals, 20,000 F98 cells in 5 µL phosphate-buffered saline (PBS) were injected over a period of 10 min. The syringe was kept in place for five min post-inoculation, and then slowly removed. The skin was sutured, and meloxicam was administered subcutaneously (1 mg/kg, 2 mg/mL) to manage post-operative pain. In the remainder of this manuscript, we refer to the inoculation day as post-inoculation day 0 (PID0).

### 2.4. In Vivo Magnetic Resonance Imaging

In 9/30 animals, in vivo 7-T micro-MRI (PharmaScan 70/16, Bruker BioSpin, Ettlingen, Germany) was acquired on PID10 to visualize tumor presence and determine the volume of the estimated abnormal mass. Animals were anesthetized with a mixture of oxygen and isoflurane (induction: 5%; maintenance: 2%) and placed in the scanner, where the isoflurane-oxygen mixture was administered through a nose cone fixed onto the animal holder. A heating pad using a warm water circuit to maintain body temperature at 37 °C was placed underneath the animal. A rat head volume coil (Bruker BioSpin, Ettlingen, Germany) was placed around the head. The respiratory rate was monitored during the entire protocol. After performing a localizer scan and optimizing the magnetic field homogeneity, a TurboRARE T2-weighted anatomical scan (TR = 3661 ms, TE = 37.1 ms, number of averages = 4, FOV = 35 × 35 mm^2^, in-plane resolution = 109 × 109 μm^2^, 600 µm slice thickness, 600 µm interslice distance, 30 slices, TA = 9′45″) was performed. Additionally, diffusion images were acquired with a spin-echo, echo-planar imaging (EPI) sequence between the olfactory bulb and the cerebellum. Diffusion-weighted acquisitions were recorded using an encoding scheme of 32 gradient directions with a b-value of 800 s/mm^2^ and with 5 b0 images. Other diffusion scanning parameters were as follows: TR = 7400 ms, TE = 24 ms, number of averages = 1, FOV = 35 × 35 mm^2^, matrix = 105 × 105, in-plane resolution = 333 × 333 μm^2^, 600 μm slice thickness, 600 μm interslice distance, 30 slices, 18.5 min acquisition time.

In the remaining 21 animals, no in vivo MRI could be performed, as these animals were implanted with vagus nerve stimulation electrodes that were assembled in a connector block on the head of the animals for another simultaneous experiment, and that were incompatible with MRI.

DTI data processing and analyses were performed using ExploreDTI v4.8.6 (in Matlab 2017b) to extract MD images.

### 2.5. Transcardial Perfusion

All animals (*n* = 30) were euthanized at PID10 (immediately after the in vivo MRI for GB01–GB09) with an overdose of sodium pentobarbital (200 mg/kg, i.p., Vétoquinol S.A./N.V., Aartselaar, Belgium). Next, animals were transcardially perfused with phosphate-buffered saline (PBS) followed by 4% paraformaldehyde (PFA). The brains were isolated and post-fixated in 4% PFA for 24 h.

### 2.6. Ex Vivo Magnetic Resonance Imaging

Ex vivo MR images of all isolated brains (*n* = 30) were acquired on the same 7-T micro-MRI system, using a transmit body volume coil (Rapid Biomedical, Germany) and an actively decoupled rat head surface coil (Rapid Biomedical, Germany) to receive the signal. The brains were removed from the 4% PFA solution, patted dry and transferred to a 10 mL tube containing fomblin (Sigma-Aldrich, Overijse, Belgium) that was inserted in the coils. After performing a localizer scan and optimizing the magnetic field homogeneity, TurboRARE T2-weighted anatomical scans (TR = 3700 ms, TE = 37.1 ms, number of averages = 12, FOV = 20 × 20 mm^2^, in-plane resolution = 62.5 × 62.5 μm^2^, 600 µm slice thickness, 600 µm interslice distance, 30 slices, TA = 29′36″) were performed.

Fomblin is a proton-free fluid that is used in ex vivo MRI to reduce magnetic susceptibility artifacts, as it produces no MRI signal and has a similar magnetic susceptibility to tissue [35].

### 2.7. Histology

Brains were removed from the fomblin solution, patted dry and rinsed three times in PBS to remove the fomblin solution, in order to avoid interference with subsequent histological analyses. Next, brains were cryoprotected using increasing sucrose solutions (10–20–30%) at 4 °C. Subsequently, the brain tissue was patted dry again, orientated in a cryomold (30 × 22 × 20 mm; Fisher Scientific, Merelbeke, Belgium) filled with O.C.T. compound (VWR international, Haasrode, Belgium), and frozen in isopentane (2-methylbutane, Sigma-Aldrich, Overijse, Belgium) and liquid nitrogen (−196 °C). The embedded frozen brain tissue was stored at −80 °C until further processing.

For cryosectioning, the embedded frozen brain tissue was removed from the cryomold and fixated on the cryostat holder with O.C.T. compound, allowing sectioning from the back. Coronal sections of 20 μm throughout the tumor were made using a cryostat (Leica CM3050S, Germany). Every 30th section (every 600 µm) was mounted directly on a glass slide, and cresyl violet staining was performed. Some additional 20 µm-thick sections containing tumor tissue were also mounted on glass slides for immunohistochemical analysis.

With the slice thickness of 20 µm and the interslice gap of 580 µm, we aimed to mimic the MRI situation in which slices were imaged every 0.6 mm as closely as possible. However, it is important to note that the slice thickness used for MRI was 600 µm, whereas this slice thickness was only 20 µm for histological analyses.

### 2.8. Volume Calculations

**Figure 1:**
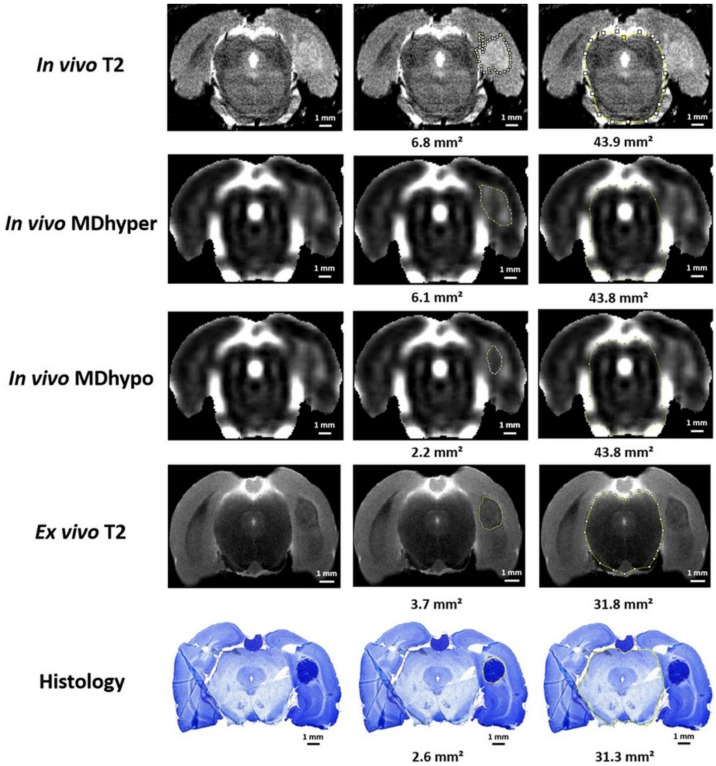
In vivo MRI, ex vivo MRI and histological analysis in GB04. In this figure, examples of in vivo T2w, in vivo MD, ex vivo T2w and histological images are shown in the first column. In the second column, the region of the estimated abnormal mass or tumor tissue is marked on these images. The third column shows the delineation of the brainstem. Calculated areas of the marked regions are indicated underneath the images. Note that exact one-by-one comparisons of slices from different modalities are not possible, as slices of the exact same locations for in vivo MRI, ex vivo MRI and histology were not available and as the slice thickness on MRI (0.6 mm) does not match the slice thickness of histological slices (20 µm).

Volumes of the estimated abnormal mass on magnetic resonance images and tumor tissue volumes on cresyl violet-stained histological slices were determined using ImageJ (National Institutes of Health, Bethesda, MD, USA). On each in vivo or ex vivo T2w image or histological slice with visible lesions, the region of the estimated abnormal mass or tumor tissue around the inoculation site in the right-sided temporal lobe was marked, and the surface area was determined (Figure 1, 2nd column). For in vivo MD images, the surface areas of the hypointense core (Figure 1, 2nd column, in vivo MDhypo) and the hyperintense rim (Figure 1, 2nd column, in vivo MDhyper)—which also included the hypointense core—were both determined. Volumes per rat brain were calculated by summating the surface areas and by multiplying the total surface area by 0.6 mm (0.6 mm slice thickness for MRI, or 20 µm slice thickness + 580 µm interslice gap for histological slices).

Localizing the abnormal mass and distinguishing between the hyperintense outer rim and the hypointense core were less straightforward with in vivo MD images. We could easily identify the abnormal mass, but an exact separation of the hyperintense and hypointense regions was more challenging. Boundaries were estimated purely visually, by delineating the most abrupt change in signal intensity from the hypointense core to the hyperintense rim. Delineations were all performed by a single observer who applied the same delineation criteria for every image.

As brain tissue shrinks during fixation (applicable for ex vivo MRI) and subsequent freezing (applicable for histology), correction factors were calculated for ex vivo MRI and histology to compare them to in vivo MRI (T2w or MD images). In six animals (GB04 to GB09), in vivo MRI, ex vivo MRI and histology were performed, and the images of these six animals were used to estimate mean correction factors for ex vivo MRI and histology. Correction factors were determined based on the shrinkage of the brainstem, as this region was the most straightforward to delineate on all modalities. Brainstem surface areas were determined on all ex vivo T2w images per animal with an abnormal mass, and the average was taken. This was performed for all six animals, and the mean ex vivo T2w brainstem surface area was calculated. Brainstem surface areas were then determined on corresponding in vivo images per animal, and the average (of an equal number of slices) was taken. This was again performed for all six animals, and the mean in vivo brainstem surface area was calculated. To obtain a mean ex vivo correction factor, the mean in vivo brainstem surface area was divided by the mean ex vivo brainstem surface area. Similarly, a mean histological correction factor was calculated by determining a mean histological brainstem surface area, based on brainstem surface areas on all histological images with tumor tissue among the six animals, and a mean in vivo brainstem surface area, based on corresponding in vivo T2w images and by dividing the mean in vivo brainstem surface area by the mean histological brainstem surface area. The ex vivo and histological correction factors were used to correct estimated ex vivo and histological volumes by multiplying the estimated ex vivo and histological volumes by the corresponding factor.

The term “tumor mass” will be used in this manuscript to indicate the region with dense GB tumor cells, without changes in the peritumoral zone. Of course, the living tumor involves both the tumor mass and a peritumoral zone including edema, inflammatory cells, and infiltrative GB cells.

### 2.9. Immunohistochemical Analyses

Immunohistochemical staining for glial fibrillary acidic protein (GFAP) and vimentin, for ionized calcium-binding adapter molecule 1 (Iba-1) and for aquaporin-4 (AQP4) were performed on the brain slices from eight animals (GB22, GB24–GB30) to highlight reactive astrocytes, activated microglia and edema, respectively. Briefly, 20 µm-thick mounted slices were first treated with 0.5% and 1% H_2_O_2_ in PBS for 30 and 60 min, respectively, to block endogenous peroxidase activity. Secondly, sections were incubated for 45 min with a blocking solution containing PBS, 0.2% Triton X-100 and 0.4% fish-skin gelatin. Next, primary antibodies were applied for 1 h at room temperature at the following dilutions: rabbit anti-GFAP (1:1000; DAKO, Heverlee, Belgium), mouse anti-vimentin (1:250; DAKO, Heverlee, Belgium), rabbit anti-Iba1 (1:500; Abcam, Cambridge, UK) and mouse anti-AQP4 (1:500; Abcam, Cambridge, UK). After extensive washing, the sections were incubated for 1 h with goat anti-rabbit-Alexa fluor 594 (1:1000; DAKO, Heverlee, Belgium) and goat anti-mouse-Alexa fluor 488 (1:1000; DAKO, Heverlee, Belgium) secondary antibodies. After thorough rinsing, DAPI (1 µg/mL) was applied for 1 min for nuclear staining. A droplet of Fluoroshield histology mounting medium (Sigma-Aldrich, Bornem, Belgium) was applied to the slices and slices were coverslipped.

Ten regions of interest (ROIs) were determined in the ipsilateral hemisphere, of which five in the tumor mass and five in the peritumoral region, as well as five ROIs in the contralateral brain hemisphere. A fixed threshold was applied using ImageJ, and the integrated density (the thresholded area multiplied by the mean gray value) for each ROI was calculated and averages of the five ROIs per brain region were determined.

### 2.10. Statistical Analyses

All statistical analyses were performed in SPSS (version 26, Chicago, IL, USA) with the level of statistical significance set at 0.05. Unless otherwise stated, data are expressed as means and standard error of the mean (SEM). Paired-samples T tests were used to compare the calculated volumes between the different imaging modalities (in vivo T2, in vivo MD, ex vivo T2 and histology). A bivariate Pearson correlation was used to explore possible correlations between in vivo MRI, ex vivo MRI and histological data. A Kruskal–Wallis test was used to compare the integrated density of immunohistochemical markers between ROIs in the peritumoral region, the tumor mass and contralateral brain hemisphere.

## 3. Results

### 3.1. Calculated and Corrected Volumes

Table 1 summarizes the volumes of the estimated abnormal mass delineated on in vivo T2w and in vivo MD images, as well as corrected volumes of the estimated abnormal mass on ex vivo T2w images and corrected histological tumor volumes. The correction factors, to compensate for shrinking during tissue processing, that were applied for comparing ex vivo T2w images and histological images were 1.21 and 1.26, respectively. Examples of delineations of the estimated abnormal mass on MRI and of tumor volume on histology are shown in Figure 1.

### 3.2. Volumes of the Estimated Abnormal Mass on In Vivo T2w Images Were Significantly Larger Than Histological Tumor Volumes

**Figure 2:**
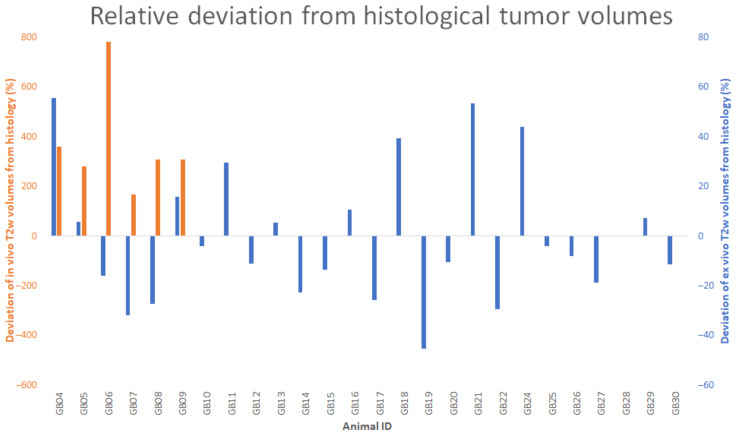
Plot of volume deviations on in vivo and ex vivo T2w images compared to histology. In this plot, the relative deviation of volumes of the estimated abnormal mass on in vivo and ex vivo T2w images compared to histological tumor volumes is shown, with a mean relative deviation of 368.2% and 21.0%, respectively. Note the difference in the range of the axes. This figure illustrates that there is no trend in the difference between volumes of the estimated abnormal mass on ex vivo T2w images and histological tumor volumes, whereas the volumes of the estimated abnormal mass on in vivo T2w images tend to be much bigger than the histological tumor volumes.

The estimated abnormal mass on in vivo T2w images (16.2 ± 1.7 mm^3^) was on average four times larger (t(5) = 13.6951, *p* < 0.001; Figure 2, orange) than histological tumor volumes (4.2 ± 0.9 mm^3^). Similarly, a significant difference in volumes (t(8) = 9.940, *p* < 0.001) was found between the estimated abnormal mass on in vivo T2w (15.3 ± 1.3 mm^3^) and ex vivo T2w (4.5 ± 0.6 mm^3^) images. Note that volumes of the estimated abnormal mass on the in vivo T2w images were only available in nine animals (Table 1, GB01–GB09). Ex vivo T2w images were available in all of these animals (Table 1, GB01–GB09), whereas histological images were only available in six out of these animals (Table 1, GB04–GB09), leading to averages from nine volumes of estimated abnormal mass on ex vivo T2w images and six histological tumor volumes for comparison to volumes of estimated abnormal mass on in vivo T2w images.

### 3.3. Volumes of the Estimated Abnormal Mass on Ex Vivo T2w Images Were More Comparable to Histological Tumor Volumes

**Figure 3:**
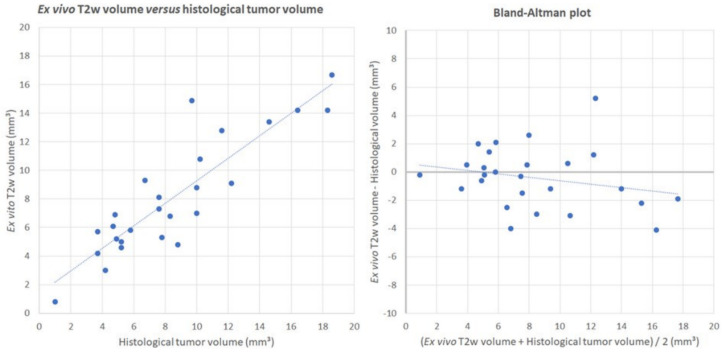
Correlation and difference between ex vivo T2w MRI and histology. These plots visualize the correlation and the difference between volumes of the estimated abnormal mass on ex vivo T2w images and histological tumor volumes. The left plot visualizes the significant strong correlation between the volumes calculated on ex vivo MRI and histology (r = 0.884; *p* < 0.001). The right plot visualizes the difference between both modalities and indicates that there is no systematic difference between volume estimations from both modalities.

No significant differences (t(25) = 0.986; *p* = 0.333) were found when comparing volumes of the estimated abnormal mass on ex vivo T2w images (8.1 ± 0.8 mm^3^) with histological tumor volumes (8.5 ± 0.9 mm^3^), with a mean deviation of volumes on ex vivo T2w images from histology of 21.0% (Figure 2, blue). A statistically significant strong correlation between volumes calculated on ex vivo MRI and histology (r = 0.884; *p* < 0.001) was found (Figure 3). Due to problems with histology in three animals (Table 1, GB01–GB03), histological tumor volumes could only be compared with ex vivo MRI in 26 out of 29 animals (Table 1, GB04–GB22, GB24–GB30).

### 3.4. Hyperintense Regions on In Vivo MD Images Are Similar to Regions of the Estimated Abnormal Mass on In Vivo T2w Images

Volumes of the hyperintense rim on in vivo MD images (14.5 ± 1.9 mm^3^) did not significantly differ (t(8) = 0.643; *p* = 0.538) from the estimated abnormal mass on in vivo T2w images (15.3 ± 1.3 mm^3^).

### 3.5. Comparison of Hypointense Regions on In Vivo MD Images and Tumor Volumes Determined on Ex Vivo MRI and Histology

A significant difference was found (t(8) = 4.071, *p* = 0.004) between volumes of the hypointense regions on in vivo MD images (2.9 ± 0.5 mm^3^) and volumes of the estimated abnormal mass on ex vivo T2w images (4.5 ± 0.6 mm^3^), as well as a significant difference (t(5) = 3.134, *p* = 0.026) between volumes of the hypointense core (2.8 ± 0.7 mm^3^) and histological tumor volumes (4.2 ± 0.9 mm^3^), with mean deviations in volume of 66.4% and 72.7%, respectively.

### 3.6. Immunohistochemical Characterization of the Tumor Mass and the Peritumoral Region

**Figure 4:**
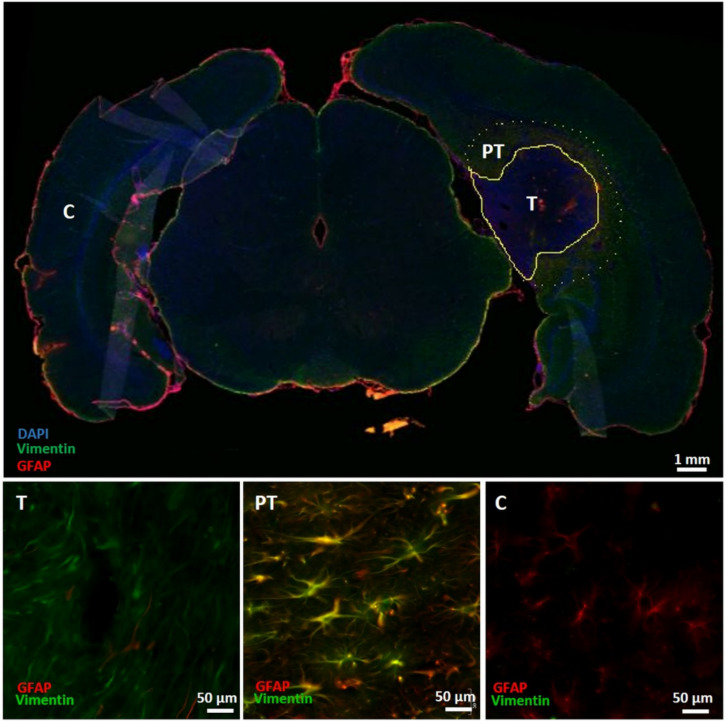
Immunohistochemical analysis for GFAP and vimentin. The upper panel shows an overview slice with GFAP, vimentin and DAPI expression. The tumor mass (T) contains more densely packed cells compared to neighboring non-tumor tissue, as demonstrated by DAPI-positive nuclei. This zone is delineated by a yellow line. Around the tumor mass, a rim of GFAP/vimentin double-positive cells can be observed, and this peritumoral area (PT) was delineated by a yellow dashed line. The lower panel shows ROIs in the different zones. T: ROI in the tumor mass with GFAP and vimentin overlay; most cells in tumor tissue are only vimentin-positive. PT: ROI in the peritumoral region with GFAP and vimentin overlay; GFAP and vimentin labeling are both dense in peritumoral tissue, with numerous double-positive cells (yellow). C: ROI in the contralateral healthy brain tissue with GFAP and vimentin overlay; astrocytes in the contralateral hemisphere were mainly vimentin-negative, whereas astrocytes were vimentin-positive in and around tumor tissue. T—tumor mass; PT—peritumoral region; C—contralateral healthy brain tissue.

**Figure 5:**
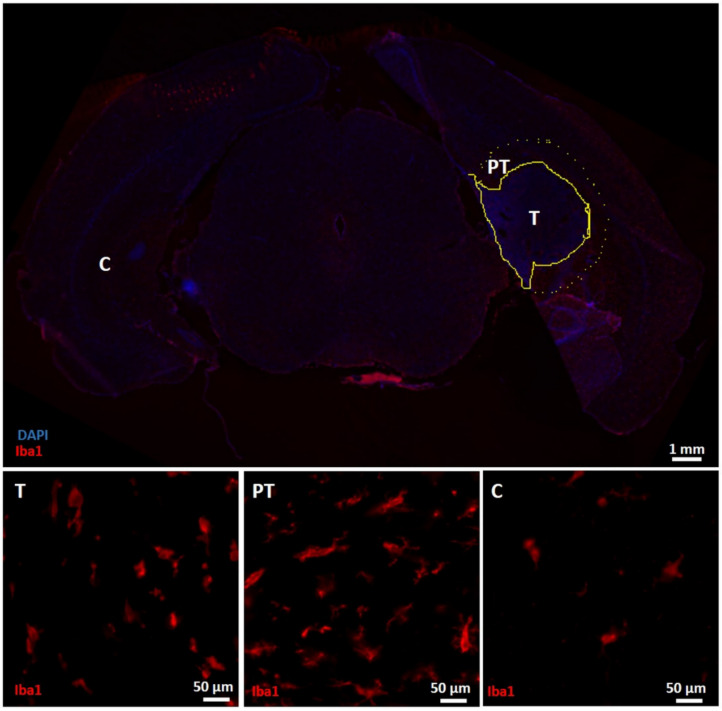
Immunohistochemical analysis for Iba1. The upper panel shows an overview slice with Iba1 and DAPI expression. The tumor mass (T) is delineated by a yellow line. Around the tumor mass, a peritumoral area (PT) was delineated by a yellow dashed line. The lower panel shows ROIs in the different zones. T: ROI in the tumor mass with Iba1 staining; infiltration of granular Iba1-positive cells can be observed. PT: ROI in the peritumoral region with Iba1 staining; numerous Iba1-positive cells with more processes were found around the tumor mass. C: ROI in the contralateral healthy brain tissue with Iba1 staining; Iba1-positive cells are identified, but less present compared to the tumor mass and especially the peritumoral region. T—tumor mass; PT—peritumoral region; C—contralateral healthy brain tissue.

**Figure 6:**
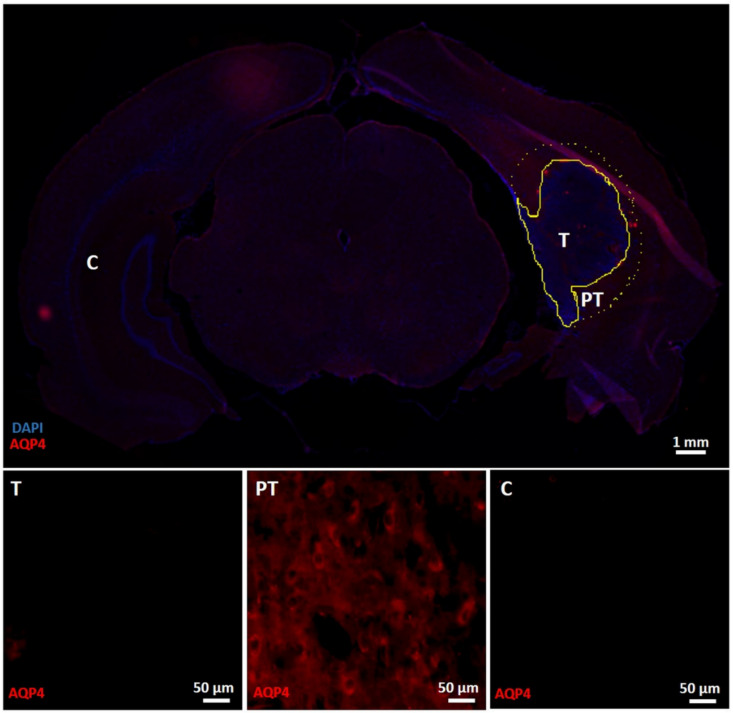
Immunohistochemical analysis for AQP4. The upper panel shows an overview slice with AQP4 and DAPI expression. The tumor mass (T) is delineated by a yellow line. Around the tumor mass, a peritumoral area (PT) was delineated by a yellow dashed line. The lower panel shows ROIs in the different zones. T: ROI in the tumor mass with AQP4 staining; AQP4 staining intensity is increased in tumor mass compared to the contralateral healthy brain tissue (C). PT: ROI in the peritumoral region with AQP4 staining; a significant increase in AQP4 staining intensity is observed in the peritumoral regions. C: ROI in the contralateral healthy brain tissue with AQP4 staining; AQP4 staining intensity is low. T—tumor mass; PT—peritumoral region; C—contralateral healthy brain tissue.

The staining intensity of GFAP, vimentin, Iba1 and AQP4 (Figure 4, Figure 5 and Figure 6) was significantly different between ROIs in the tumor mass, the peritumoral region and the contralateral healthy brain tissue (Kruskal–Wallis test, χ^2^(2) = 7.875, *p* = 0.019; χ^2^(2) = 15.005, *p* = 0.001, χ^2^(2) = 11.285, *p* = 0.004 and χ^2^(2) = 16.640, *p* < 0.001, respectively). Post hoc pairwise tests revealed significant increases in the staining intensity of vimentin, Iba1 and AQP4 in peritumoral regions compared to the contralateral healthy brain tissue (*p* = 0.001, *p* = 0.002 and *p* < 0.001, respectively). Numerous GFAP/vimentin double-positive cells aligned at the tumor border (Figure 4, PT). The staining intensity of vimentin and AQP4 was also significantly increased in the tumor mass compared to the contralateral hemisphere (*p* = 0.019 and *p* = 0.014, respectively). Moreover, the staining intensity of GFAP was significantly higher in peritumoral regions compared to the tumor mass (*p* = 0.024), where fewer GFAP-positive cells were observed in the tumor mass (Figure 4, T) compared to the contralateral healthy brain tissue (Figure 4, C). Many Iba1-positive cells can also be observed in the tumor mass (Figure 5, T), but this is less pronounced compared to the peritumoral regions (Figure 5, PT). Iba1-positive cells in peritumoral regions seemed to be somewhat hypertrophic and have thicker and shorter processes, and most Iba1-positive cells in the tumor mass appeared as smaller rounded or amoeboid cells, compared to Iba1-positive cells with more and thinner branching processes in the contralateral healthy brain tissue. Almost no AQP4 positivity was observed in the contralateral healthy brain tissue (Figure 6, C).

## 4. Discussion

MRI plays a crucial role in the diagnosis and treatment monitoring of GB. Currently, both in clinical practice and in preclinical GB animal models, brain tumors are primarily characterized by their appearance on T1w images, before and after contrast enhancement, and on T2w images [18]. These sequences also highlight abnormal regions around the tumor besides the tumor cells themselves and, therefore, the exact delineation of GB tumors is challenging [20]. Caution is, thus, warranted when using these techniques to investigate the effect of novel therapies on tumor volumes.

The present study shows that the estimated abnormal mass on in vivo T2w images was approximately four times larger in volume compared to histological tumor volumes. Volumes of the estimated abnormal mass on ex vivo T2w images were more comparable to these latter volumes. Therefore, estimation of the abnormal mass on ex vivo T2w images most likely reflects the dense tumor mass, whereas the estimated abnormal mass on in vivo T2w images includes the peritumoral zone as well. Immunohistochemical staining for vimentin and Iba1 revealed significant increases in staining intensity in peritumoral regions compared to the contralateral healthy brain tissue, as well as a slight increase in the staining intensity of GFAP. Dense GFAP and vimentin labeling in the brain parenchyma surrounding the tumor mass reflect the presence of reactive astrocytes [36]. Tumor-associated astrocytes can be activated by neighboring glioma cells, and reactive astrocytes promote proliferation, invasion, and treatment resistance of GB via a complex interaction (involving gap junctions, ion channels and transporters, chemokines, and cytokines) with the TME [37]. Vimentin expression was also increased in the tumor mass, whereas GFAP expression in the tumor mass was even slightly lower compared to its expression in the contralateral hemisphere. As vimentin expression is an early event in normal glial differentiation, while GFAP only appears in later stages, this indicates that the majority of tumor cells in these GB tumors were neoplastic astrocytes in a dedifferentiated state [36]. The increased staining intensity of Iba1 around the tumors indicates an accumulation of glioma-associated microglia/macrophages (GAMs) in tumor-bearing hemispheres [38,39]. These GAMs are believed to have pro-tumorigenic roles in tumor progression, as these cells are recruited by glioma cells, thereby establishing an immunosuppressive tumor environment and stimulating cell proliferation and the migration of glioma cells. GAMs may also promote angiogenesis [40]. Moreover, many Iba1-positive cells were found in the tumor mass, indicating the infiltration of microglia in the tumor mass. Iba1-positive cells in the tumor mass and peritumoral regions have distinct shapes compared to Iba1-positive cells in the contralateral healthy brain tissue. Swelling of the cells and the shortening and thickening of the processes in peritumoral microglia may indicate an activated state, whereas smaller, round or amoeboid-shaped microglia in the tumor mass may indicate a reactive state [41].

DTI quantifies water diffusion in vivo, and its derived metric MD is a measure of the overall magnitude of diffusion [42]. In in vivo MD images in the present study, GB tumors were displayed as two regions with distinct intensities: a hyperintense rim and a core of a more hypointense signal. Lower MD values and, thus, a more hypointense signal on MD images might indicate high cellularity in the tumor mass, whereas higher MD values resulting in a hyperintense signal might represent peritumoral edema and necrotic tumor areas [29]. Immunohistochemical analyses also revealed an increased staining intensity of AQP4 in peritumoral areas that may be associated with the presence of peritumoral edema. Increases in AQP4 expression surrounding the bulk tumor mass were previously shown in female Fischer rats bearing F98 tumors by Engelhorn et al. [43], and they suggested that this increased expression by swollen astrocytes may reflect a final attempt to restore the extracellular balance of fluids. In our study, the hyperintense rim on in vivo MD images seemed to reflect areas of the estimated abnormal mass on in vivo T2w images. Together with the increased staining intensity of AQP4 in peritumoral regions, this validates that edema causes hyperintensity on in vivo T2w images and is, thus, also included in tumor volume estimations on in vivo T2w images. Volumes of the hypointense core are most likely indicative of the tumor mass. However, significant differences were observed between the volumes of the hypointense core on in vivo MD images and the volumes of the estimated abnormal mass on ex vivo T2w images or histological tumor volumes. The transition from the hypointense core to the hyperintense rim on our in vivo MD images occurred gradually, making an exact visual distinction difficult. The lower resolution on in vivo MD images compared to in vivo and ex vivo T2w images may cause partial volume effects, which may explain both the smaller volumes of the hypointense core and the vague transition zone. 

A gradient of ADC values, derived from DWI, was found in the region of peritumoral edema in GB patients: ADC values increased from the region close to the enhancing tumor to the area near the normal-appearing white matter. This gradient may represent a gradient in neoplastic cells as GB tumors consist of a core tumor mass surrounded by a penumbra of invasive malignant cells that decrease in number toward the periphery [44]. As DTI is derived from DWI and demands magnetic gradients in at least six directions, this gradient could also be expected on MD images. It would be interesting to further explore the transition zone on in vivo MD images and correlate this to the expression of immunohistochemical markers, and also one-by-one with the tumor tissue extent on cresyl violet-stained histological slices. Therefore, it would be useful to use a marker, such as bronze iridescent acrylic paint, that is appropriate for fiducial marker creation in MRI at 7 T, both pre- and post-fixation, and is also visible on histology [45]. This would allow for careful slice selection and more exact comparison of the estimated abnormal mass on in vivo T2w, in vivo MD images and ex vivo T2w images with cresyl violet and immunohistochemical staining on histological slices. This way, it will be possible to correlate hypointense zones on in vivo MD images to the tumor mass and infiltrations on cresyl violet-stained histological slices, and to characterize the transition zone on in vivo MD images and the estimated abnormal mass on in vivo and ex vivo T2w images, using immunohistochemical markers for reactive astrocytes, inflammation and edema. Moreover, the use of a marker that can be detected both on MRI and histology may allow the better determination of correction factors for the comparison of volumes between the different modalities.

The peritumoral zone, including inflammatory cells, edema and infiltrative GB cells, is an essential part of the tumor, as these changes may also contribute to GB progression. Therefore, in vivo MRI remains the gold standard for treatment monitoring in GB. However, we believe that, from an experimental point of view, it might be interesting to differentiate the effect of treatments on the tumor mass from the effect on GB infiltration, peritumoral edema and inflammatory response. The use of three modalities may allow a better understanding of the GB pathophysiology, and how treatments impact the tumor. In vivo MRI provides a general representation of the tumor mass and peritumoral tissue changes, ex vivo MRI allows a delineation of the tumor mass, and histology allows the visualization and characterization of the tumor mass, the peritumoral GB cell infiltrations and inflammatory cells. As a rat model for GB-associated epilepsy [16] was used in the current study, further optimization of in vivo MRI protocols, in combination with extensive histological analyses, may allow achieving a more detailed characterization of the model and help to understand the process of epileptogenesis in GB. The implantation of MRI-compatible electrodes would allow researchers to continuously record EEG for the detection of epileptic seizures and study their relation to glioma-induced anatomical and functional changes on follow-up (functional) MRI scans during GB progression.

The rat model for glioma-associated epilepsy used male Fischer rats only. Male rats were chosen to minimize confounding effects (hormonal cycle in female rats). Therefore, we also only used male rats during this study. In future studies, it will be necessary to study the seizure occurrence and characterize the GB tumors in female rats as well, to make the results fully applicable for therapeutic screening use and translation to humans.

## 5. Conclusions

This study shows that, by combining imaging techniques using different structural scales, a better understanding of the pathophysiology of GB can be obtained. The results of the present study show that hyperintense regions on in vivo T2w images not only represent the tumor mass but also include peritumoral changes, such as perifocal edema including reactive astrocytes and activated microglia, as indicated by a comparison of in vivo T2w images with in vivo MD images, histological and immunohistochemical analyses. However, a more careful slice selection and more exact comparison of slices will be needed to confirm this. Caution is warranted when using in vivo T2w images for treatment monitoring in preclinical GB models, as the effect of treatments on the tumor mass may be confounded by effects on tumor-related brain tissue changes. Ex vivo T2w images allow a proper volume estimation of the tumor mass. Therefore, these scans might be used for end-stage tumor volume estimations when brain tissue is needed for other analyses, not allowing further histological processing. Moreover, as in vivo MD images display GB tumors as two regions with distinct intensities—a hypointense core and a hyperintense rim, probably reflecting tumor mass and edema, respectively—these images might be useful in the future to discriminate between the tumor mass and peritumoral edema in vivo.

## Figures and Tables

**Table 1:** Tumor volume estimations from the different modalities. This table summarizes the volumes of the estimated abnormal mass delineated on in vivo and ex vivo MRI, and tumor volumes delineated on cresyl violet-stained histological slices. Corrected volumes are shown of the estimated abnormal mass on ex vivo T2w images and histological tumor volumes for comparison with in vivo MRI. GB023 was not included in the table nor for the analyses, as no tumor could be detected.

Rat ID	Volume of Estimated Abnormal Mass on In Vivo T2w Images (mm^3^)	Volume of the Hyperintense Rim on In Vivo MD Images (mm^3^)	Volume of the Hypointense Core on In Vivo MD Images (mm^3^)	Corrected Volume of Estimated Abnormal Mass on Ex Vivo T2w Images (mm^3^)	Corrected Histological Tumor Volume (mm^3^)
GB01	15.0	17.5	3.5	7.3	
GB02	16.2	13.7	3.8	5.3	
GB03	9.2	11.6	2.1	3.6	
GB04	16.8	16.0	2.6	5.7	3.7
GB05	18.8	19.7	4.4	5.2	4.9
GB06	8.9	5.3	0.5	0.8	1.0
GB07	20.9	23.6	4.9	5.3	7.8
GB08	17.0	8.4	1.4	3.0	4.2
GB09	14.9	14.8	2.9	4.2	3.7
GB10				5.0	5.2
GB11				6.1	4.7
GB12				4.6	5.2
GB13				10.8	10.2
GB14				14.2	18.3
GB15				14.2	16.4
GB16				12.8	11.6
GB17				9.1	12.2
GB18				9.3	6.7
GB19				4.8	8.8
GB20				16.7	18.6
GB21				14.9	9.7
GB22				7.0	10.0
GB24				6.9	4.8
GB25				7.3	7.6
GB26				13.4	14.6
GB27				6.8	8.3
GB28				5.5	5.8
GB29				8.1	7.6
GB30				8.8	10.0

## Data Availability

The datasets supporting the conclusions of this article are stored on local servers of Ghent University and will be made available by the corresponding author upon reasonable request.
